# The study of protein recruitment to laser-induced DNA lesions can be distorted by photoconversion of the DNA binding dye Hoechst

**DOI:** 10.12688/f1000research.17865.2

**Published:** 2019-04-11

**Authors:** Verena Hurst, Susan M. Gasser

**Affiliations:** 1 Friedrich Miescher Institute for Biomedical Research, Maulbeerstrasse 66, Basel, CH-4058, Switzerland; 2 Faculty of Natural Sciences, University of Basel, Basel, CH-4056, Switzerland

**Keywords:** Photoconversion, Hoechst, DAPI, UV laser, DNA repair

## Abstract

A commonly used approach for assessing DNA repair factor recruitment in mammalian cells is to induce DNA damage with a laser in the UV or near UV range and follow the local increase of GFP-tagged proteins at the site of damage. Often these measurements are performed in the presence of the blue DNA dye Hoechst, which is used as a photosensitizer. However, a light-induced switch of Hoechst from a blue-light to a green-light emitter will give a false positive signal at the site of damage.  Thus, photoconversion signals must be subtracted from the overall green-light emission to determine true recruitment. Here we demonstrate the photoconversion effect and suggest control experiments to exclude false-positive results.

## Abbreviations

ATM: *ataxia telangiectasia mutated* protein kinase, DAPI: 4', 6-diamidino-2-phenylindole; UV: ultraviolet light; U2OS: human bone osteosarcoma epithelial cells; GFP: green fluorescent protein; 53BP1: tumor suppressor p53-binding protein 1; XRCC1: x-ray repair cross-complementing protein 1; FEN-1: Flap endonuclease 1; PARP-1: poly [ADP-ribose] polymerase 1; KU70/XRCC6: 5'-deoxyribose-5-phosphate lyaseKu70/X-ray repair cross-complementing protein 6, LigIII: DNA ligase 3, MDC1: mediator of DNA damage checkpoint 1; PCNA: proliferating cell nuclear antigen, RPA: replication protein A SMARCA5: SWI/SNF-related matrix-associated actin-dependent regulator of chromatin subfamily A member 5

## Introduction

A variety of DNA binding dyes, such as DAPI and Hoechst can change their optical properties upon exposure to light ^1, 2^. This process, termed photoconversion, can occur during multicolor fluorescence microscopy and may lead to false-positive signals ^2, 3^.

Upon exposure to UV or to low pH, the emission spectra of DAPI and Hoechst shift from the blue to the green wavelength with detectable signals in the yellow, orange and red wavelengths ^1, 2, 4, 5^. This shift makes the signal indistinguishable from the emission of other standardly used fluorescent proteins such as GFP. An experimenter expecting that the DNA dyes emit in the blue range can misinterpret the green signal as that arising from another probe in the sample. This risk has been raised previously ^1, 3, 6^, yet the artefact is rarely controlled for.

With respect to these findings, a microscopy setup like the one used to study the localization of repair proteins to a near UV/UVlaser-induced zone of DNA damage can be particularly problematic. Very commonly, cell nuclei are sensitized with Hoechst and a restricted part of the nucleus is exposed to a UV/near UV laser. The protein of interest is detected in the green channel thanks either to its fusion to GFP or else through an antibody labelled with a green light-emitting fluorophore. Unfortunately, photoconversion of the DNA dye is rarely checked ^7– 11^. Here we will illustrate the problem and suggest necessary controls.

## Results

**Figure 1.**
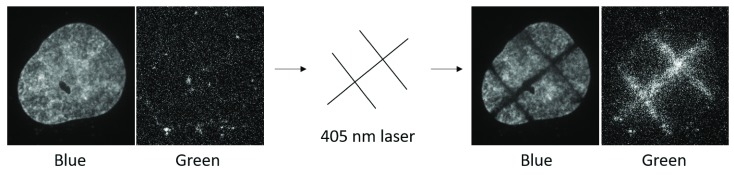
Representative U2OS cell nucleus before and after 405 nm laser-induced photoconversion of Hoechst.

To study the recruitment of a potential DNA damage related protein, we made use of a previously established protocol in which cell nuclei are sensitized with Hoechst, DNA damage is induced with a near UV laser, and the recruitment of a protein of interest is measured over time by fluorescence microscopy. Unexpectedly, cells stained with Hoechst that did not express any GFP-tagged protein showed a similar increase in the green channel at the laser damage site ( Figure 1), as cells expressing the GFP-tagged protein. The detected increase in signal was not due to protein recruitment to the damage site, since there was no GFP-tagged protein in the cell. Moreover, in cells expressing the GFP-tagged protein that were not stained with Hoechst, there was no increase in signal intensity at the laser damage site. This demonstrates conclusively that the increase in fluorescence in the green channel was a false-positive result. Raw images are available on figshare ^12^.

## Discussion

We illustrate here that one should avoid exposing DAPI or Hoechst to a strong UV/near UV laser if one is imaging green light emitting probes such as GFP or a secondary antibody coupled to fluorescein/Alexa488, because photoconverted Hoechst and DAPI strongly emit in the same channel.

We note that the laser power used varies among studies. Our study uses high laser power in order to demonstrate the photoconversion effect. Nonetheless, even smaller amounts of photoconverted dye will alter the signal intensity measured. Therefore, quantification of a control sample is essential to any study, especially if the behavior of the studied protein upon damage is not previously known. A recent study detected photoconversion and adapted the experimental setup accordingly ^13^.

An alternative means to visualize the nucleus is to introduce a fluorescently tagged protein that localizes to the nuclear rim, assuming that it does not interfere with the experimental process. The outline of the nucleus can also be determined by means of a transmitted light image. When employing Hoechst as a sensitizing agent, we suggest using the lowest possible dye concentration and laser power, and to combine these with probes/secondary antibodies that emit in a range that is easily separable from that of photoconverted Hoechst, for instance, a far red emitter ^1^. Nonetheless, accurate quantitation of the signal of the fluorescent protein of interest requires normalization to a background control, which requires that one performs the laser experiment on Hoechst-stained but otherwise native cells lacking the tagged protein. The control signal should be acquired with the same channel and exposure conditions, as used for the experimental probe.

It is important to note that it is possible to avoid photosensitization through exogenous DNA-binding compounds altogether, in the study of DNA damage factors. The compounds are sometimes added in order to alter the type of damage generated. Two commonly studied UV products are cyclobutane pyrimidine dimers (CPD) and 6-4 photoproducts (6-4PP), which are generated by UV-C (100-280 nm ^14^) or UV-A irradiation (315-380 nm ^14^) ^9, 15^. However, UV-A exposure causes oxidative lesions and double strand breaks (DSBs) as well ^15– 17^, while UV-C does not induce DSBs ^9, 15– 17^. Intriguingly, in the presence of sensitizing agents such as BrdU or Hoechst, both UV-A and visible light (≤390 nm) generated mostly DSB and oxidative lesions ^17^. Moreover, the addition of Hoechst followed by 405 nm light led to the increased generation of the typical UV photoproduct CPD ^18^. This lesion, however, can also be studied without Hoechst and UV-A/C irradiation.

A further argument for performing repair studies without Hoechst are the effects of the reagent on transcription and genomic stability. Hoechst binds primarily in the DNA minor groove and therefore competes with other minor groove binding proteins like TATA-box transcription factors ^19^. Thus, besides photoconversion, Hoechst treatment can have side effects such as altered transcription ^20^, the inhibition of DNA synthesis and an accumulation of mutations ^21^.

Several studies show that visible light is sufficient to cause DNA breaks ^22^ and that DNA repair factors or checkpoint kinases, such as pATM ^22^, RPA ^22^, 53BP1 ^15, 23, 24^, XRCC1 ^15, 22^, FEN-1 ^15^, PCNA ^22^, LigIII ^22^, PARP-1 ^15^, KU70 ^15^, MDC1 ^24^, and SMARCA5 ^24^, are recruited to sites of damage without previous sensitization. A study recently monitored the kinetics of recruitment and turnover of 70 proteins at UV-induced DNA damage sites without sensitizing agents, and modeled these results mathematically ^25^.

Finally, in addition to particular situations in which one induces local damage with a laser, the photoconversion of DAPI from blue to green and red can occur during standard dual color microscopy on fixed samples ^2, 3^. To minimize artefacts one should be careful about the order in which dyes are observed, starting always with the longer wavelengths ^3^.

## Methods

U2OS cells (a gift from Prof. Primo Leo Schaer, Department of Biomedicine, University of Basel) were incubated with 1.5 µg/ml Hoechst 33342 (Thermo Fisher Scientific, H1399) for at least 30 minutes prior to photoconversion. Photoconversion was induced with a VisiFRAP module (Visitron) mounted on the backport of the microscope and equipped with a 405 nm laser (Toptica, illumination power at the objective 12.8 mW, ≥1ms/pixel). Confocal images were acquired with an Olympus IX81 microscope equipped with a PlanApo 100x/1.45 TIRFM oil objective, a CSU-X1 scan-head (Yokogawa), an Evolve 512 EMCCD camera (Photometrics), a 491nm laser (Cobolt Calypso 100), a 488/568 dichroic (Semrock Di01-T488/568-13x15x0.5), a band-pass 525/40 emission filter (Semrock FF01-525/40-25) and controlled with the Visiview Software (Visitron). Images in Figure 1 show maximum intensity projections of stacks ^12^ covering 7 µm. 

## Data availability

Raw images of the stacks taken during this study are available on figshare. DOI: https://doi.org/10.6084/m9.figshare.7583960.v2 ^12^.

Data are available under the terms of the Creative Commons Zero "No rights reserved" data waiver (CC0 1.0 Public domain dedication).

## Media

The three available avi files, C1 green, C2 blue and composite, represent a time series of maximum intensity projections showing the 405nm laser-induced emission change of the DNA binding dye Hoechst from the blue to the green region of the visible spectrum. Under live conditions, a Hoechst-stained cell nucleus was irradiated with 405 nm laser light along a predefined pattern. A time series of image stacks was acquired (25 equally spaced time points over 65s, stacks covering 7-µm sample depth) in two channels (C1 “green”: 491/525 nm, C2 “blue”: 405/450 nm). DOI: https://doi.org/10.6084/m9.figshare.7583960.v2 ^12^.
