# Supplementary material for: The study of protein recruitment to laser-induced DNA lesions can be distorted by photoconversion of the DNA binding dye Hoechst
Source: F1000Res. 2019 Apr 11;8:104. Originally published 2019 Jan 25. [Version 2] doi: 10.12688/f1000research.17865.2 (PMC6392149; doi:10.12688/f1000research.17865.2)
Supplement: Peer review history

# Peer review: Reviewer response for version 2

**DOI:** 10.5256/f1000research.20618.r47073

**Published:** 2019-05-30

**Authors:** Jerzy Dobrucki

**Affiliations:** 1 Department of Cell Biophysics, Faculty of Biochemistry, Biophysics, and Biotechnology, Jagiellonian University, Krakow, Poland

A new section (" Several studies show that visible light is sufficient to cause DNA breaks...")    addresses the issue of an apparent ability to induce DNA damage, including DNA breaks, by visible light (i.e. approx. 400-750nm) in the absence of exogenous photosensitisers. While the notion is correct, three of the four cited papers do not support it.

 Recruitment of 53BP1 to DNA lesions is backed by three references that are inappropriate for the following reasons:

 Kong et al. used several pulsed lasers (and 405 nm cw) and exceedingly high light intensities to induce local DNA damage. The damage was so extensive that it caused local change in optical properties of cytoplasm – this was revealed by phase contrast microscopy. Such damage must have been much more severe than just an induction of a few DSBs which would result in a subsequent recruitment of repair factors, including 53BP. The authors discuss several consequences of exposing  cells to the laser beams they used and mention even formation of plasma. Induction of a wide spectrum of cellular lesions, including DNA lesions of many types, in the experimental systems used by Kong et al. has no simple mechanistic explanation and does not constitute a piece of evidence testifying to an ability of visible light to cause biologically relevant DNA damage in a form of DSBs, which would elicit physiologically relevant DNA damage response. Thus quoting this report in the context of inducing DNA damage by visible light is misleading.

 The Kong et al. paper is also quoted in the context of recruitment of FEN-1, PARP-1, and Ku70. Here also this reference is not appropriate for the reasons explained above.

 Carvalho et al. used 405nm laser light (at 100% output as stated by the authors), without using exogenous photosensitisers. Since 405nm light is just on the edge of the visible range, and this wavelengths induces typical damage characteristic for UV (photoproducts, pyrimidine dimers as a result of direct absorption of a photon by DNA, and secondary damage) quoting this paper does not support the notion that visible light can induce DNA damage without sensitisation.

 Smeenk et al. induced “DSB-containing tracks (1.5mm  width)… with a Mira mode locked   Ti:Sapphire laser (lambda 800nm, pulse length 5200 fs, repetition rate 576 MHz, output power 80 mW)”. 800nm is not visible light. Although the authors do not state this directly, the intensity of light they used must have resulted in two photon excitation. Thus, in their experiments the cells were exposed locally to infrared radiation of high intensity, and experienced two photon absorption phenomena equivalent to 400 nm excitation – again not typical visible light but the wavelength on the border between UV and visible range. For this reason this reference is misleading. 

 The Smeenk et al. paper is also quoted in the context of MDCC1 and SAMARCA5 recruitment and is inappropriate for the reasons explained above.

 Finally, the new section on DNA damage induced by visible light seems to have listed a number of repair factors associated with double-strand breaks, but has not mentioned single-strand breaks. Blue light-induced recruitment of XRCC1, a factor which is involved in repair of SSBs, has been demonstrated in the reference number 22 (as well as two other papers from our laboratory).

 Rewriting the section about DNA damage induced by visible light in the absence of exogenous photosensitisers is advised as this is an important argument in favour of studying recruitment of repair factors under conditions that permit physiologically relevant damage and permit physiological repair processes.

 Hope this helps.

I have read this submission. I believe that I have an appropriate level of expertise to confirm that it is of an acceptable scientific standard, however I have significant reservations, as outlined above.

# Peer review: Reviewer response for version 2

**DOI:** 10.5256/f1000research.20618.r47075

**Published:** 2019-04-18

**Authors:** Sophie E. Polo

**Affiliations:** 1 Epigenetics & Cell Fate Centre, Paris Diderot University, Paris, France

The revised version addresses the reviewers' comments.

I have read this submission. I believe that I have an appropriate level of expertise to confirm that it is of an acceptable scientific standard.

# Peer review: Reviewer response for version 1

**DOI:** 10.5256/f1000research.19537.r43604

**Published:** 2019-02-26

**Authors:** Jerzy Dobrucki

**Affiliations:** 1 Department of Cell Biophysics, Faculty of Biochemistry, Biophysics, and Biotechnology, Jagiellonian University, Krakow, Poland

The paper “The study of protein recruitment…” by Hurst and Gasser is a very useful and timely technical report. It touches upon an important but often overlooked methodological aspect of studies of recruitment of repair factors to DNA lesions. When unnoticed, photoconversion of UV-excited DNA fluorescent probes can constitute a pitfall leading to incorrect interpretation of imaging data.

  General comments:

 “UV-induced lesions”

 In the title and the text of this report the authors use a term “UV-induced DNA lesions”. The use of this term is misleading, since UV-induced lesions are generally defined as pyrimidine dimers and photoproducts. However, the authors refer to DNA lesions inflicted by 405nm light (which is not UV, see below) in the presence of a DNA-bound fluorescent dye Hoechst, and this leads to induction of a host of various lesions, not only “UV-induced”. When Hoechst 33342 has been introduced into live cells and exposed to 405nm focused laser light, it is (marginally) excited and acts as a photosensitizer. This leads to induction of oxidative damage and DNA breaks, i.e. not typical UV-induced lesions.

 “UV laser”

 The term ‘UV laser’ is used throughout the manuscript. This term appears incorrect, since the authors used a laser emitting 405nm wavelength light. By definition, 405 nm is visible light (and indeed it is readily visible by human eye, as opposed to UVA, UVB or UVC). I suggest to refer to the Toptica laser, which the authors used, as ‘blue laser”, as in the paper [Kong et al. 2009] which the authors cite.

 Hoechst photosensitization vs DNA labeling

 Fluorescent DNA probe Hoechst is described in two disguises in this paper – as a photosensitizer and as a counterstain. These two roles and the ensuing problems are not clearly distinguished and explained in the paper. If Hoechst or DAPI are added to live cells prior to microirradiation with a focused beam of light of 405nm wavelength, the dye acts as a photosensitizer (as correctly stated in Results). However, in the Abstract the authors state that “the measurements are performed in the presence of the blue intercalating dye Hoechst or DAPI which is used to label nuclear DNA”. This statement appears incorrect for two reasons: 1. Hoechst is usually added to live cells in order to photosensitise and yield massive, readily detectable damage, not to just label DNA (if Hoechst were to be used as a label to mark DNA, it could be added after microirradiation); 2. Hoechst and DAPI are not DNA intercalators, they are rather minor groove binders (the mode of binding is complex and depends on a number of factors, including the type of DNA and a DNA/dye ratio).

 Minor comments:

 It is unclear what the authors mean by referring to photoconversion of Hoechst, DAPI and VybrantDyeCycle and, in the same sentence and context, to blinking of YoYo described as “a change in optical properties”. What change of optical properties of YoYo do the authors have in mind?

 The authors state (in Discussion): “As an alternative nuclear marker, we suggest employing a fluorescently tagged protein that localizes at the nuclear periphery and does not interfere with the experimental process.”     Why use such a complicated approach? It is much simpler to detect a transmitted light image, preferably using phase contrast or Nomarski interference contrast, and mark the outline of the nucleus in image overlay.

  The authors state: “However, there is evidence showing that DNA sensitization prior to laser exposure is not required:...” This is true, but again the way the authors put it is somewhat confusing and they refer to research in which UVA as well as visible light was used. Indeed, photosensitisation by exogenous DNA-binding compounds is never required – regardless of the type of light, UV or VIS. Moreover, adding a photosensitizer to the experimental system influences the type of damage. Thus, I suggest to distinguish two cases:

1. using UV-excited dyes and UV or near-UV (405nm) light, and
2. using dyes excited by visible light and visible light excitation.

 

 In the case (1) the exciting light (UV or 405) will induce typical UV damage (PP, PD). Adding a photosensitizer prior to microirradiation will result in photodynamic effect, and cause induction of more types of lesions, and more extensive damage. There is vast literature about the action of UV alone and photodynamic effect type I and II. Some reference to this field of knowledge should be made in this paper.

 Contrary to general belief, in the case (2) the exciting visible light alone, without any exogenous photosensitisers, will also induce DNA damage – single- and double-strand breaks, and recruitment of repair factors (Solarczyk et al., 2012, DNA Repair ^1^).

 In summary, indeed various types of DNA damage can be induced without adding photosensitisers to cells prior to microirradiation, not only when UV, or 405 nm light is used, but also when visible light is applied. I suggest to clarify these facts in the paper.

  A few inaccurate statements should be straightened out:

- “A common approach used to assess DNA repair factor binding in mammalian cells is to induce DNA damage with a UV laser and follow the movement of GFP-tagged proteins to the site of damage”. To be precise, induction of (local) DNA damage by a focused laser beam is used to detect recruitment of repair factors, but not their binding per se.
- “Movement of GFP-tagged proteins to the site of damage”. Movement of GFP-tagged proteins is not detected (this can be done by FCS) – only local increase of a concentration of a fusion protein is detected, and this arises from recruitment.

 The authors state:

 „exposing DAPI or Hoechst to a strong UV laser” - light intensities and doses of energy delivered to the exposed region of the nucleus (area?) should be given.

  Full information (dose) is lacking but it appears that the authors used an excessive power of 405nm light (12.8 mW at objective). DNA damage, especially in the presence of a photosensitizer, can be expected when using only microJ of energy (microW in the laser beam, seconds of exposure). This means that the photoconversion the authors describe most likely would have been less prominent, had a lower intensity and dose of energy been used. Applying excessive energy during microirradiation leads to such an extensive damage that relevant physiological studies may be impossible (Note that some images in the paper [Kong et al.] show microirradiation tracks in phase contrast images; the power which was used in this study was very high). I suggest that the authors state that the intensities (and doses of energy) they used may have been too high to induce DNA damage on the level encountered under typically encountered physiological conditions.

 Typo:

 “Here will illustrate the problem and suggest necessary controls.”  Here we will.

 Hope this helps.

I have read this submission. I believe that I have an appropriate level of expertise to confirm that it is of an acceptable scientific standard, however I have significant reservations, as outlined above.

## References

[1] SolarczykKJZarębskiMDobruckiJW: Inducing local DNA damage by visible light to study chromatin repair. *DNA Repair (Amst)*.2012;11(12) : 10.1016/j.dnarep.2012.09.008 996-1002 10.1016/j.dnarep.2012.09.008 23089313

# Response

**Published:** 2019-03-31

**Authors:** Verena Hurst

**Affiliations:** Friedrich Miescher Institute Basel, Switzerland

A=Authors, R=Reviewer

A: Thank you for sharing your expertise. Your comments helped to significantly increase the quality of the article.

R: “UV-induced lesions”

In the title and the text of this report the authors use a term “UV-induced DNA lesions”. The use of this term is misleading, since UV-induced lesions are generally defined as pyrimidine dimers and photoproducts. However, the authors refer to DNA lesions inflicted by 405nm light (which is not UV, see below) in the presence of a DNA-bound fluorescent dye Hoechst, and this leads to induction of a host of various lesions, not only “UV-induced”. When Hoechst 33342 has been introduced into live cells and exposed to 405nm focused laser light, it is (marginally) excited and acts as a photosensitizer. This leads to induction of oxidative damage and DNA breaks, i.e. not typical UV-induced lesions.

A: We added information on the types of damage generated at different wavelengths +- photosensitizing agents.

R:“UV laser”

The term ‘UV laser’ is used throughout the manuscript. This term appears incorrect, since the authors used a laser emitting 405nm wavelength light. By definition, 405 nm is visible light (and indeed it is readily visible by human eye, as opposed to UVA, UVB or UVC). I suggest to refer to the Toptica laser, which the authors used, as ‘blue laser”, as in the paper [Kong et al. 2009] which the authors cite.

A: You are right. We corrected that.

R: Hoechst photosensitization vs DNA labeling

Fluorescent DNA probe Hoechst is described in two disguises in this paper – as a photosensitizer and as a counterstain. These two roles and the ensuing problems are not clearly distinguished and explained in the paper. If Hoechst or DAPI are added to live cells prior to microirradiation with a focused beam of light of 405nm wavelength, the dye acts as a photosensitizer (as correctly stated in Results). However, in the Abstract the authors state that “the measurements are performed in the presence of the blue intercalating dye Hoechst or DAPI which is used to label nuclear DNA”. This statement appears incorrect for two reasons: 1. Hoechst is usually added to live cells in order to photosensitise and yield massive, readily detectable damage, not to just label DNA (if Hoechst were to be used as a label to mark DNA, it could be added after microirradiation); 2. Hoechst and DAPI are not DNA intercalators, they are rather minor groove binders (the mode of binding is complex and depends on a number of factors, including the type of DNA and a DNA/dye ratio).

A: We corrected and clarified that point. Actually using Hoechst helps identify the placement of the laser beam.

R: Minor comments:

It is unclear what the authors mean by referring to photoconversion of Hoechst, DAPI and VybrantDyeCycle and, in the same sentence and context, to blinking of YoYo described as “a change in optical properties”. What change of optical properties of YoYo do the authors have in mind?

A: In order to focus on Hoechst and DAPI we removed the comments on the other two dyes.

R: The authors state (in Discussion): “As an alternative nuclear marker, we suggest employing a fluorescently tagged protein that localizes at the nuclear periphery and does not interfere with the experimental process.”     Why use such a complicated approach? It is much simpler to detect a transmitted light image, preferably using phase contrast or Nomarski interference contrast, and mark the outline of the nucleus in image overlay.

A: We and other labs standardly use fluorescent labels of the nuclear envelope without complications. We are not sure transmitted light images are compatible with rapid time lapse imaging since filters have to be changed but we mentioned this possibility in the text.

R: The authors state: “However, there is evidence showing that DNA sensitization prior to laser exposure is not required:...” This is true, but again the way the authors put it is somewhat confusing and they refer to research in which UVA as well as visible light was used. Indeed, photosensitisation by exogenous DNA-binding compounds is never required – regardless of the type of light, UV or VIS. Moreover, adding a photosensitizer to the experimental system influences the type of damage. Thus, I suggest to distinguish two cases:

1. using UV-excited dyes and UV or near-UV (405nm) light, and
2. using dyes excited by visible light and visible light excitation.

 

In the case (1) the exciting light (UV or 405) will induce typical UV damage (PP, PD). Adding a photosensitizer prior to microirradiation will result in photodynamic effect, and cause induction of more types of lesions, and more extensive damage. There is vast literature about the action of UV alone and photodynamic effect type I and II. Some reference to this field of knowledge should be made in this paper.

A: We have added a section discussing the types of damage generated under different conditions.

R: Contrary to general belief, in the case (2) the exciting visible light alone, without any exogenous photosensitisers, will also induce DNA damage – single- and double-strand breaks, and recruitment of repair factors (Solarczyk et al., 2012, DNA Repair ^1^).

A: We noticed this phenomenon in our own experiments, and have now commented upon this in the text and have cited the reference suggested.

R: In summary, indeed various types of DNA damage can be induced without adding photosensitisers to cells prior to microirradiation, not only when UV, or 405 nm light is used, but also when visible light is applied. I suggest to clarify these facts in the paper.

 A few inaccurate statements should be straightened out:

- “A common approach used to assess DNA repair factor binding in mammalian cells is to induce DNA damage with a UV laser and follow the movement of GFP-tagged proteins to the site of damage”. To be precise, induction of (local) DNA damage by a focused laser beam is used to detect recruitment of repair factors, but not their binding per se.

A: True. Corrected.

- R: “Movement of GFP-tagged proteins to the site of damage”. Movement of GFP-tagged proteins is not detected (this can be done by FCS) – only local increase of a concentration of a fusion protein is detected, and this arises from recruitment.

A: Corrected.

R: The authors state:

„exposing DAPI or Hoechst to a strong UV laser” - light intensities and doses of energy delivered to the exposed region of the nucleus (area?) should be given.

Full information (dose) is lacking but it appears that the authors used an excessive power of 405nm light (12.8 mW at objective). DNA damage, especially in the presence of a photosensitizer, can be expected when using only microJ of energy (microW in the laser beam, seconds of exposure). This means that the photoconversion the authors describe most likely would have been less prominent, had a lower intensity and dose of energy been used. Applying excessive energy during microirradiation leads to such an extensive damage that relevant physiological studies may be impossible (Note that some images in the paper [Kong et al.] show microirradiation tracks in phase contrast images; the power which was used in this study was very high). I suggest that the authors state that the intensities (and doses of energy) they used may have been too high to induce DNA damage on the level encountered under typically encountered physiological conditions.

A: See response to Anna Fortuny, who raised this issue as well. Now we state that we use high laser power in order to demonstrate the effect and provide further information on the laser conditions. Furthermore, we refer to a study detecting and minimizing such an effect in their setup.

R: Typo:

“Here will illustrate the problem and suggest necessary controls.”  Here we will.

A: Corrected.

R: Hope this helps.

A: Yes. Thank you!

# Peer review: Reviewer response for version 1

**DOI:** 10.5256/f1000research.19537.r43603

**Published:** 2019-02-11

**Authors:** Sophie E. Polo, Anna Fortuny

**Affiliations:** 1 Epigenetics & Cell Fate Centre, Paris Diderot University, Paris, France

In this research note, Hurst & Gasser highlight Hoechst photoconversion as a potential caveat in UV laser damage experiments. The UV-induced switch of Hoechst (or DAPI) from blue to green may indeed give a false positive signal in the green channel.

 The following points need to be addressed:

-  The authors refer to a “strong UV laser”. It is not clear which conditions are required for DAPI/Hoechst photoconversion (laser power, exposure time) and how they compare to classical exposure times for DAPI visualization and to UV laser settings for laser damage induction. It is important to clarify this point to determine if we face a marginal or a general problem. 
- The authors should be more specific when referring to UV laser or UV light. They should specify “UVA”, as other UV wavelengths such as UVC, also used to introduce local DNA damage (Dinant  et al.,2007 ^1^), may not have the same effect.
- They could suggest using BrdU instead of Hoechst to pre-sensitize cells to UVA light as done in a number of studies (e.g. Lukas et al.,2003 ^2^). 
- The authors illustrate the problem with GFP-tagged proteins but the issue would be similar with YFP-tagged proteins. They explain that far-red emission is compatible with photoconverted DAPI/Hoechst. How about red emission?
- There are 55 files on figshare, which seems excessive, and they are difficult to navigate through so in the end it is not very useful. 
- Result section: “cells expressing the GFP-tagged protein (Figure 1)”. These cells are not shown on the figure and which GFP-tagged protein is it?

We have read this submission. We believe that we have an appropriate level of expertise to confirm that it is of an acceptable scientific standard, however we have significant reservations, as outlined above.

## References

[1] DinantCde JagerMEssersJvan CappellenWAKanaarRHoutsmullerABVermeulenW: Activation of multiple DNA repair pathways by sub-nuclear damage induction methods. *J Cell Sci*.2007;120(Pt 15) : 10.1242/jcs.004523 2731-40 10.1242/jcs.004523 17646676

[2] LukasCFalckJBartkovaJBartekJLukasJ: Distinct spatiotemporal dynamics of mammalian checkpoint regulators induced by DNA damage. *Nat Cell Biol*.2003;5(3) : 10.1038/ncb945 255-60 10.1038/ncb945 12598907

# Response

**Published:** 2019-03-31

**Authors:** Verena Hurst

**Affiliations:** Friedrich Miescher Institute Basel, Switzerland

Thank you very much for your comments! Version 2 of this research note accommodates the changes that you suggested unless stated otherwise.

-  The authors refer to a “strong UV laser”. It is not clear which conditions are required for DAPI/Hoechst photoconversion (laser power, exposure time) and how they compare to classical exposure times for DAPI visualization and to UV laser settings for laser damage induction. It is important to clarify this point to determine if we face a marginal or a general problem. 

Indeed, we use higher laser power than used in most studies in order to demonstrate the photoconversion effect. The purpose of this research note is to increase the awareness of the phenomenon and highlight that a negative control is particularly essential in this experimental setup. Even small amounts of photoconverted dye can change image quantitation and may alter apparent recruitment dynamics. We do think that this effect is particularly relevant to the study of uncharacterized proteins. Explorative studies may tempt the researcher to vary laser power and exposure time because the degree of damage required for protein recruitment is not known. This bears the risk of a false positive result. A preventive measure is to generate the damage without using Hoechst.

We now cite a study in which photoconversion was detected and minimized, indicating the relevance of this note.

A recent report on photoconversion in standard multiple color microscopy applications specifies exposure times for photoconversion ^1^.

- The authors should be more specific when referring to UV laser or UV light. They should specify “UVA”, as other UV wavelengths such as UVC, also used to introduce local DNA damage (Dinant  et al.,2007 ^1^), may not have the same effect.

We added a section on the type of damage generated +-sensitizing agents and cited Dinant et al.

- They could suggest using BrdU instead of Hoechst to pre-sensitize cells to UVA light as done in a number of studies (e.g. Lukas et al.,2003 ^2^). 

Now we mention BrdU as a sensitizing agent and specify which type of damage is generated in its presence (double strand breaks ^2^). However, we favor omission of sensitizing agents altogether.

- The authors illustrate the problem with GFP-tagged proteins but the issue would be similar with YFP-tagged proteins. They explain that far-red emission is compatible with photoconverted DAPI/Hoechst. How about red emission?

We have used GFP in our setup and therefore mainly discuss GFP. However, according to the publications we cited the effect may apply to YFP and other proteins with emission in the orange and near red as well ^1,3^. This is now mentioned.

- There are 55 files on figshare, which seems excessive, and they are difficult to navigate through so in the end it is not very useful. 

We were asked to deposit the raw data on figshare. As mentioned in our note we also deposited avi files of timelapse MIPs in both channels as well as a channel merge. These files are are easy to handle.

- Result section: “cells expressing the GFP-tagged protein (Figure 1)”. These cells are not shown on the figure and which GFP-tagged protein is it?

We changed the position of the reference to Fig. 1 in this sentence to avoid implying that we will show cells expressing a GFP-tagged protein.

Rather than naming the protein we want to highlight that this type of false-positive result can apply to any GFP-tagged protein tested, which is not recruited to the damage site.

References

1) Karg, T. J. & Golic, K. G. Photoconversion of DAPI and Hoechst dyes to green and red-emitting forms after exposure to UV excitation. *Chromosoma* **127**, 235-245, doi:10.1007/s00412-017-0654-5 (2018).

2) Ferrando-May, E. *et al.* Highlighting the DNA damage response with ultrashort laser pulses in the near infrared and kinetic modeling. *Front Genet* **4**, 135, doi:10.3389/fgene.2013.00135 (2013).

3) Zurek-Biesiada, D., Kedracka-Krok, S. & Dobrucki, J. W. UV-activated conversion of Hoechst 33258, DAPI, and Vybrant DyeCycle fluorescent dyes into blue-excited, green-emitting protonated forms. *Cytometry A* **83**, 441-451, doi:10.1002/cyto.a.22260 (2013).

# Peer review: Reviewer response for version 1

**DOI:** 10.5256/f1000research.19537.r43632

**Published:** 2019-01-29

**Authors:** Vincent Dion

**Affiliations:** 1 UK Dementia Research Institute, Cardiff University, Cardiff , UK

This short report by Hurst and Gasser exposes an important experimental detail that is often, but not always, overlooked: Hoechst-sensitized cells irradiated with UV light produce a signal in the same region of the spectrum as GFP. Consequently, without the proper controls, it may lead to false positives. That is, that it may be falsely concluded that GFP-tagged proteins are recruited to laser-induced damage.

 In the discussion, they point out potential controls to that would prevent an experimenter to make this mistake. Specifically, they propose leaving out Hoechst or DAPI altogether, using the appropriate control cells that do not express the GFP-tagged protein of interest, using another nuclear marker, such as a protein localizing to the nuclear periphery, or using a different fluorescent protein. Those are all good ways of getting around the reported problem. I would also add the use of a UVC source (Dabin et al., 2018 ^1^).

I have read this submission. I believe that I have an appropriate level of expertise to confirm that it is of an acceptable scientific standard.

## References

[1] DabinJFortunyAPiquetSPoloSE: Live Imaging of Parental Histone Variant Dynamics in UVC-Damaged Chromatin. *Methods Mol Biol*.2018;1832: 10.1007/978-1-4939-8663-7_13 243-253 10.1007/978-1-4939-8663-7_13 30073531

# Response

**Published:** 2019-03-31

**Authors:** Verena Hurst

**Affiliations:** Friedrich Miescher Institute Basel, Switzerland

Thank you for your comments! In version 2 we have added a section discussing the different types of damage generated at different UV/VIS wavelengths.
